# Lifestyle and risk factor modification in atrial fibrillation: a European Heart Rhythm Association survey

**DOI:** 10.1093/europace/euaf075

**Published:** 2025-03-28

**Authors:** Mark T Mills, Piotr Futyma, Peter Calvert, Diego Penela, Laurent Roten, Laura Perrotta, Federico Migliore, Gregory Y H Lip, Dhiraj Gupta, Julian K R Chun

**Affiliations:** Liverpool Centre for Cardiovascular Science at University of Liverpool, Liverpool John Moores University and Liverpool Heart & Chest Hospital, Liverpool, UK; Department of Cardiology, Liverpool Heart & Chest Hospital NHS Foundation Trust, Thomas Drive, Liverpool, UK; Medical College, University of Rzeszów and St. Joseph's Heart Rhythm Center, Rzeszów, Poland; Liverpool Centre for Cardiovascular Science at University of Liverpool, Liverpool John Moores University and Liverpool Heart & Chest Hospital, Liverpool, UK; Department of Cardiology, Liverpool Heart & Chest Hospital NHS Foundation Trust, Thomas Drive, Liverpool, UK; Arrhythmology Department, IRCCS Humanitas Research Hospital, Rozzano, Italy; Department of Cardiology, Inselspital, Bern University Hospital, University of Bern, Bern, Switzerland; Department of Cardiology, Arrhythmia Unit, Careggi University Hospital, Florence, Italy; Department of Cardiac, Thoracic, Vascular Sciences and Public Health, University of Padova, Padova, Italy; Liverpool Centre for Cardiovascular Science at University of Liverpool, Liverpool John Moores University and Liverpool Heart & Chest Hospital, Liverpool, UK; Department of Cardiology, Liverpool Heart & Chest Hospital NHS Foundation Trust, Thomas Drive, Liverpool, UK; Department of Clinical Medicine, Danish Center for Health Services Research, Aalborg University, Aalborg, Denmark; Liverpool Centre for Cardiovascular Science at University of Liverpool, Liverpool John Moores University and Liverpool Heart & Chest Hospital, Liverpool, UK; Department of Cardiology, Liverpool Heart & Chest Hospital NHS Foundation Trust, Thomas Drive, Liverpool, UK; Cardioangiologisches Centrum Bethanien, Agaplesion Markus Krankenhaus, Frankfurt am Main, Germany

**Keywords:** Atrial fibrillation, Lifestyle modification, Risk factors, Comorbidity, Cardiac rehabilitation

## Abstract

**Aims:**

Lifestyle and risk factor modification (LRFM) forms a central pillar in the management of atrial fibrillation (AF). This European Heart Rhythm Association (EHRA) survey aims to assess current clinical practice regarding LRFM across EHRA countries.

**Methods and results:**

A 31-item questionnaire was developed and distributed amongst healthcare professionals via the EHRA and social media, between 23 September and 21 October 2024. Of 258 respondents from 28 countries, 39.9% reported that their healthcare system is badly or very badly designed to deliver meaningful LRFM. Risk factors that respondents felt least confident managing included psychological distress (42.2% of respondents not confident), sleep-disordered breathing (33.8%), and obesity (22.4%). Respondents estimated that 70% of patients with AF at their institution may benefit from exercise-based cardiac rehabilitation but that only 10% are referred for this. The most important barrier to cardiac rehabilitation in AF was identified as local programmes not accepting patients with AF only (42.1% of respondents). Despite 37.7% of respondents using a body mass index cut-off when deciding on catheter ablation suitability (with a mean cut-off of 36.7 ± 5.4 kg/m^2^), only 23.5% of patients with obesity are referred for formal dietary advice. Lack of patient motivation or engagement was identified as the most important barrier to weight loss (41.3% of respondents). Moreover, 89.6% of respondents routinely assess their patient's alcohol intake, whilst only 23.9% systemically assess for psychological distress and 16.5% for sleep-disordered breathing.

**Conclusion:**

Delivering comprehensive LRFM in AF poses significant challenges. Improvements to healthcare infrastructures are required to successfully implement meaningful LRFM.

## Graphical Abstract

**Graphical Abstract:**
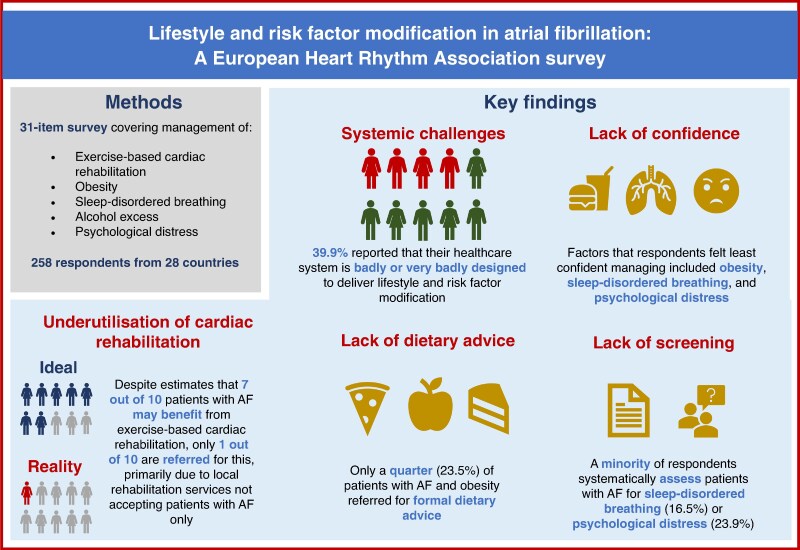

## Introduction

Atrial fibrillation (AF), the most common sustained arrhythmia, presents a significant public health challenge due to its associated morbidity, mortality, and healthcare burden.^1,2^ While drug therapy and catheter ablation^3,4^ play pivotal roles in the management of AF, growing evidence underscores the importance of lifestyle and risk factor modification (LRFM) in both the prevention and management of this condition.^5^

Risk factors such as hypertension, obesity, sleep-disordered breathing, diabetes mellitus, and excessive alcohol consumption have long been recognised as contributors to the onset and progression of AF,^6–9^ yet the potential for lifestyle changes to mitigate AF-related morbidity has only recently gained broader attention.^2,10–13^ Interventions targeting these modifiable risk factors, including physical activity, dietary improvements, weight management, and reduction in alcohol intake, have demonstrated promising effects on both the incidence and clinical course of AF.^10^

The 2024 European Society of Cardiology (ESC) guidelines for the management of AF place LRFM front and centre in the holistic management of AF, as summarised in the AF ‘CARE’ pathway, which stands for C, comorbidity and risk factor management; A, avoid stroke and thromboembolism; R, reduce symptoms by rate and rhythm control; and E, evaluation and dynamic reassessment.^14,15^ Crucially, adherence with such holistic approaches associates with improved clinical outcomes.^16,17^

In this European Heart Rhythm Association (EHRA) Scientific Initiatives Committee survey, we aimed to assess current practice and barriers to LRFM in patients with AF across ESC countries.

## Methods

### Questionnaire development and dissemination

A bespoke 31-item questionnaire was developed by the EHRA Scientific Initiatives Committee, comprising single- and multiple-choice questions and questions requiring numerical responses. The questionnaire was reviewed, edited, and approved by all co-authors, and included sections on (i) respondents’ demographics; (ii) general perceptions of LRFM in AF; (iii) the use of exercise-based cardiac rehabilitation; and (iv) individual sections on the management of obesity, sleep-disordered breathing, alcohol excess, and psychological distress in patients with AF. The full questionnaire is provided in the Supplementary Material—Supplementary material online, Appendix  *S1*.

The link to the online questionnaire was distributed to the EHRA and EHRA Young EP communities via email and also promoted via social media, between 23 September and 21 October 2024. The survey was open to all healthcare professionals managing patients with AF (including—but not limited to—clinicians, nurses, and physiologists). Response was voluntary, anonymous, and GDPR compliant.

### Statistical analysis

Continuous variables are presented as mean ± standard deviation (SD) or median and interquartile range (IQR) depending upon normality of distribution. Categorical variables were expressed as counts and percentages. Statistical analysis was performed in SPSS (version 29; IBM).

## Results

From a total of 343 responses, 85 questionnaires were blank (contained no responses to any question) leaving 258 questionnaires that were analysed further.

**Figure 1:**
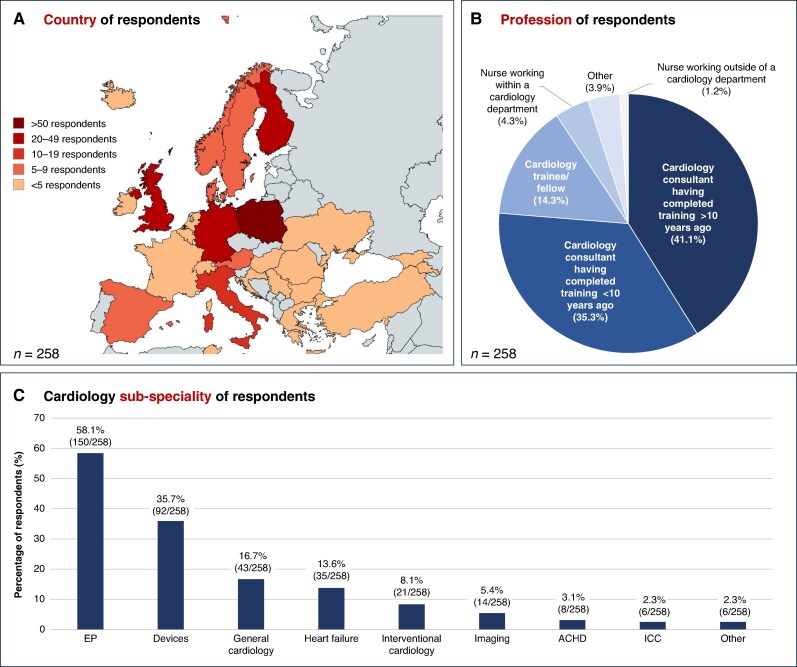
Demographics of respondents. *A*) Country of work of respondents (created with MapChart.net). *B*) Profession of respondents. *C*) Cardiology sub-speciality of respondents. ACHD, adult congenital heart disease; EP, electrophysiology; ICC, inherited cardiac conditions.

Respondents worked across 28 countries, with the number of responses per country summarised in *Figure 1A*. The five countries with the most respondents were Poland (21.3%), Finland (14.7%), United Kingdom (13.2%), Germany (10.5%), and Italy (5.0%). The professional categories of respondents included cardiology consultants having completed training greater than 10 years ago (41.1%) or within the last 10 years (35.3%), cardiology trainees or fellows (14.3%), nurses working within a cardiology department (4.3%), nurses working outside of a cardiology department (1.2%), and ‘other’ (3.9%) (*Figure 1B*). The five most common cardiology sub-specialities of respondents were electrophysiology (58.1%), devices (35.7%), general cardiology (16.7%), heart failure (13.6%), and interventional cardiology (8.1%) (*Figure 1C*). Respondents estimated managing a median of 8 patients with AF per week (IQR 8–20 patients) across all clinical settings.

### General perceptions of lifestyle and risk factor modification

**Figure 2:**
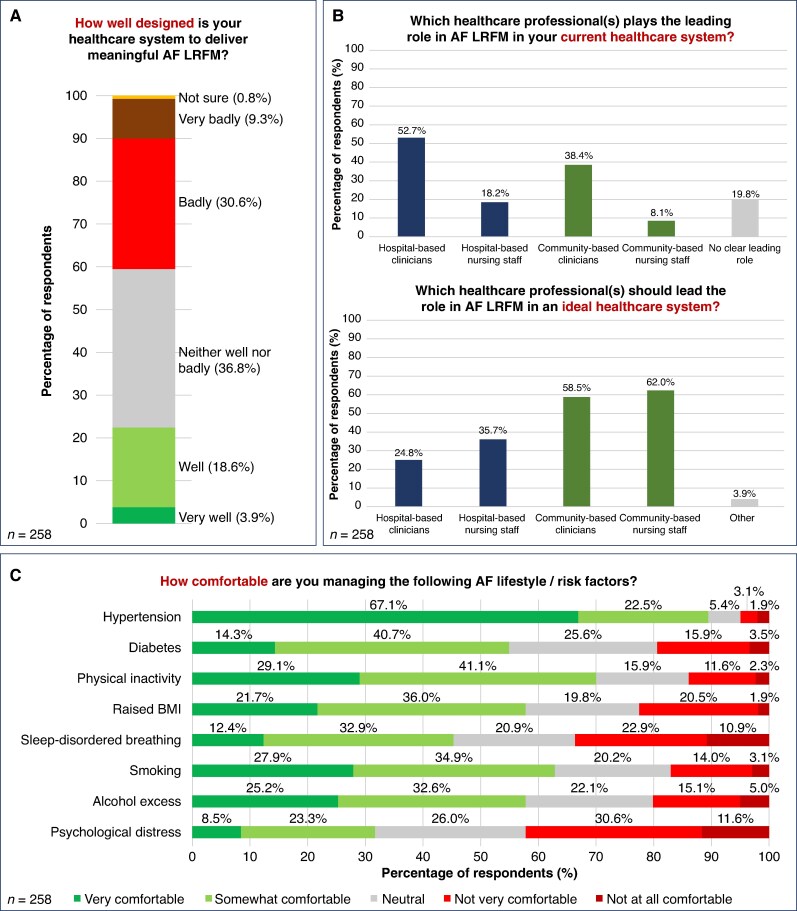
General perceptions of atrial fibrillation (AF) lifestyle and risk factor modification (LRFM). *A*) Adequacy of respondents’ healthcare systems to deliver meaningful AF LRFM. *B*) Current vs. ideal healthcare professional groups in AF LRFM. *C*) Confidence in managing AF lifestyle and risk factors.

Four out of ten respondents (39.9%) reported that their healthcare system is badly or very badly designed to deliver meaningful LRFM, with only 22.5% reporting that it is well or very well designed; 36.8% of respondents reported that their healthcare system is neither well nor badly designed (*Figure 2A*).

Respondents were asked which group(s) of healthcare professionals plays the leading role in AF LRFM in their current healthcare system and which group(s) they believed *should* play the leading role in an ideal system. In current healthcare systems, hospital-based clinicians (52.7%) and community-based clinicians (38.4%) were most commonly reported as leading AF LRFM, with hospital-based nursing staff (18.2%) and community-based nursing staff (8.1%) less often leading; 19.8% of respondents reported that no professional group currently played a leading role in AF LRFM. In contrast, in an ideal healthcare system, the majority of respondents felt that community-based clinicians (58.5%) and community-based nursing staff (62.0%) should play leading roles in AF LRFM, with only a minority of respondents believing this role was best held by hospital-based clinicians (24.8%) or hospital-based nursing staff (35.7%) (*Figure 2B*).

AF-related lifestyle and risk factors that respondents felt most confident managing included hypertension (89.6% somewhat or very comfortable), physical inactivity (70.2% somewhat or very confident), and smoking (62.8% somewhat or very comfortable). Factors that respondents felt least confident managing included psychological distress (42.2% not very or not at all comfortable), sleep-disordered breathing (33.8% not very or not at all comfortable), and excess weight and obesity (22.4% not very or not at all comfortable) (*Figure 2C*).

### Exercise-based cardiac rehabilitation

Respondents estimated that 70% (IQR 50–80%) of their patients with AF may benefit from exercise-based cardiac rehabilitation, but that only 10% (1–20%) are in fact referred for this. Only a minority of respondents (27.8%) reported that it is possible for them to refer patients with AF for exercise-based cardiac rehabilitation locally, with 58.1% of respondents reporting that this was not possible (14.1% of respondents ‘not sure’).

**Figure 3:**
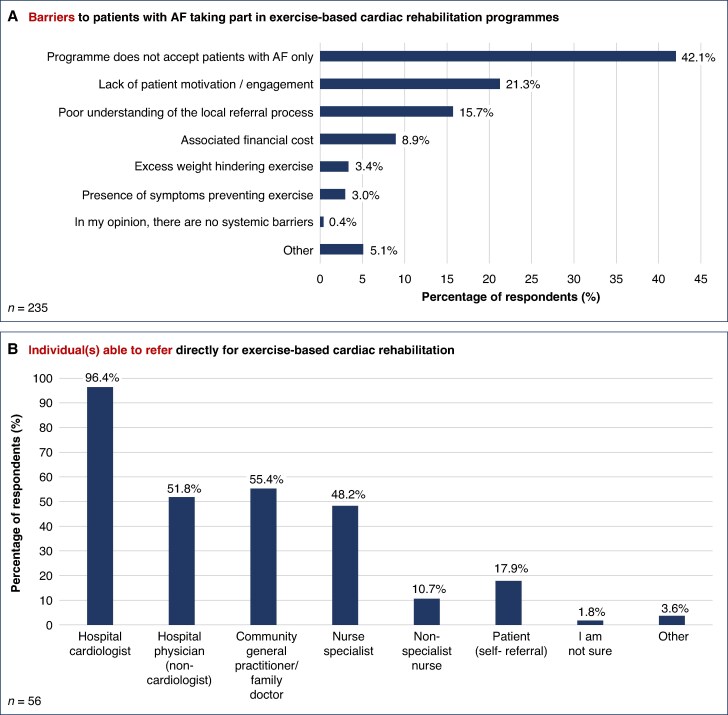
Exercise-based cardiac rehabilitation in atrial fibrillation (AF). *A*) Barriers to cardiac rehabilitation in AF. *B*) Individuals able to refer for cardiac rehabilitation in AF.

Barriers to referring patients with AF for exercised-based cardiac rehabilitation are summarised in *Figure 3A*; the three most important barriers were local cardiac rehabilitation programme not accepting patients with AF only (42.1%), lack of patient motivation/engagement (21.3%), and poor understanding of the local referral process (15.7%). In respondents with access to local cardiac rehabilitation for AF patients, individuals able to refer included hospital cardiologists (96.4%), community general practitioners (55.4%), non-cardiology hospital physicians (51.8%), and nurse specialists (48.2%), with only 17.9% of programmes accepting patient self-referrals (*Figure 3B*).

### Excess weight and obesity

**Figure 4:**
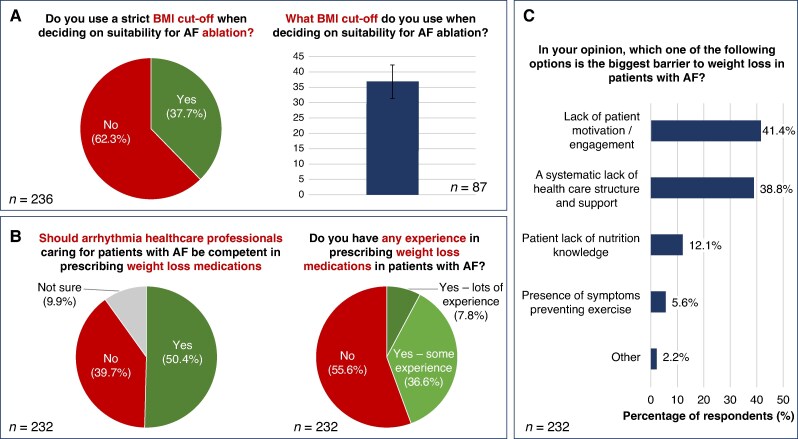
Management of raised body mass index (BMI) in atrial fibrillation (AF). *A*) Use of a BMI cut-off in patients with AF. *B*) Use of weight loss medications in patients with AF. *C*) Barriers to weight loss in patients with AF.

Respondents estimated that only 23.5% (IQR 10–50%) of their patients with AF and a raised BMI are referred for formal dietary advice (e.g. by a qualified nutritionist or dietitian). Moreover, 37.7% of respondents reported using a body mass index (BMI) cut-off when deciding on suitability for AF catheter ablation with a mean threshold of 36.7 ± 5.4 kg/m^2^ (*Figure 4A*).

Of the respondents, 50.4% believed that arrhythmia healthcare professionals should be competent in prescribing weight loss medications (e.g. orlistat, liraglutide, semaglutide). Regarding personal experience of prescribing weight loss medications in patients with AF, 7.8% of respondents reported ‘lots of experience’, 36.6% ‘some experience; and 55.6% ‘no experience’ (*Figure 4B*).

Barriers to weight loss in patients with AF are summarised in *Figure 4C*; the three most important barriers were lack of patient motivation or engagement (41.3% of respondents), a systematic lack of healthcare structure and support (38.8%), and patient lack of nutrition knowledge (12.1%).

### Sleep-disordered breathing

**Figure 5:**
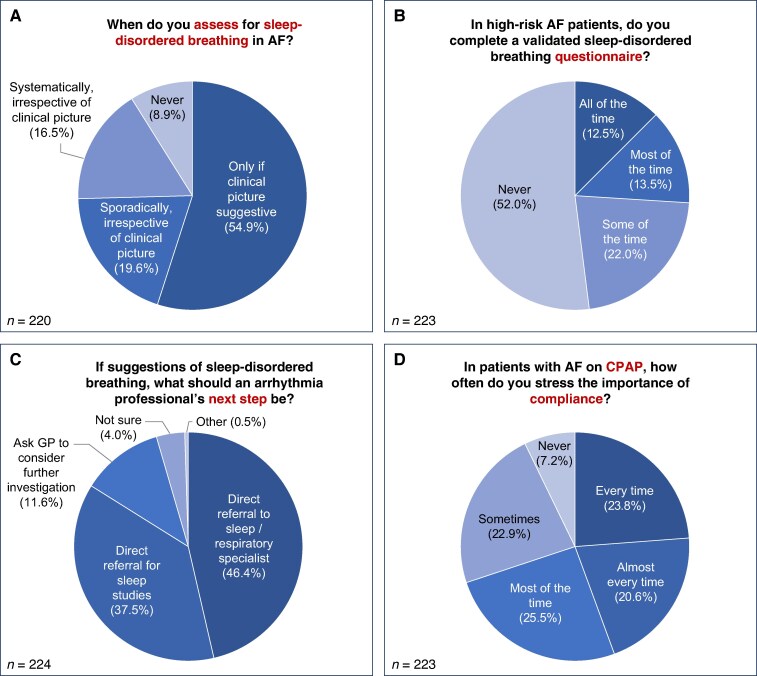
Management of sleep-disordered breathing in atrial fibrillation (AF). *A*) Assessment of potential sleep-disordered breathing in AF. *B*) Use of validated sleep-disordered breathing in high-risk patients with AF. *C*) Arrhythmia professional's next steps if symptom enquiry, clinical examination, or validated questionnaires suggestive of sleep-disordered breathing. *D*) Ensuring compliance with continuous positive airway pressure (CPAP) therapy in patients with AF.

Of the respondents, 54.9% reported only assessing for sleep-disordered breathing if the clinical picture is suggestive, while 8.9% reported never assessing. Of the remainder, irrespective of the clinical picture, 19.6% sporadically assess and 16.5% systematically assess for sleep-disordered breathing (*Figure 5A*). In patients with AF at high-risk of sleep-disordered breathing, 52.0% of respondents reported never using validated sleep-disordered breathing questionnaires, with 12.5% of respondents using these all of the time, and others using them most of the time (13.5%) or some of the time (22.0%) (*Figure 5B*).

If symptom enquiry, clinical examination, or validated questionnaires are suggestive of sleep-disordered breathing in patients with AF, 46.4% of respondents reported that an arrhythmia healthcare professional should refer to a sleep or respiratory specialist, 37.5% that they should refer directly for sleep studies, and 11.6% that they should ask the patient's general practitioner to consider further investigation if appropriate (*Figure 5C*).

In patients with AF and known sleep-disordered breathing on continuous positive airway pressure (CPAP) therapy, 23.8% of respondents always stress the importance of CPAP compliance, 20.6% almost always, 25.5% most of the time, and 22.9% some of the time, with 7.2% never stressing the importance (*Figure 5D*).

### Alcohol

The majority (89.6%) of respondents reported routinely asking patients with AF about their alcohol intake, estimating that 30% (IQR 20–50%) of this cohort drink to excess. In addition, 14.9% of respondents recommend that their patients with AF abstain from alcohol, with 56.8% recommending no more than 5 units of alcohol per week, 11.3% recommending no more than 6 to 10 units per week, and 7.7% recommending no more than 10 to 14 units a week. 5.0% of respondents reported not routinely advising on a specific alcohol limit.

Only 30.2% of respondents reported being able to refer patients with AF and alcohol excess to a dedicated alcohol reduction/cessation service in their centre.

### Psychological distress

A minority of respondents (23.9%) reported regularly assessing their patients with AF for psychological distress (4.5% every time, 5.4% almost every time, 14.0% most of the time), with 59.7% sometimes assessing, and 16.3% never assessing. When assessing for psychological distress, the majority of respondents reported using informal questioning only (72.4%), with 4.1% using validated psychological questionnaires, and 5.4% using a combination of informal questioning and questionnaires.

## Discussion

Our study is the first to report healthcare professionals’ views of LRFM in AF. The main findings of our analysis are (i) 4 out of 10 respondents reported that their healthcare system is badly or very badly designed to deliver meaningful LRFM in patients with AF; (ii) risk factors that respondents felt least confident managing included obesity, sleep-disordered breathing, and psychological distress; (iii) despite estimates that 7 out of 10 patients with AF may benefit from exercise-based cardiac rehabilitation, only 1 out of 10 are referred for this, primarily due to local rehabilitation services not accepting patients with AF only; (iv) although lack of patient motivation or engagement was reported as the most important barrier to weight loss in AF, the management of obesity appears suboptimal, with only a quarter of patients with AF and obesity referred for formal dietary advice; (v) only a minority of respondents systematically assess patients with AF for sleep-disordered breathing or psychological distress.

Overall, our findings highlight the variability and challenges in delivering AF LRFM across European centres, supporting the need for system-wide improvements and reforms to healthcare infrastructures in order to implement meaningful LRFM.

### Confidence in managing lifestyle and risk factors

Numerous studies have demonstrated the impact of LRFM in AF. In obese patients, weight loss ≥10% of body weight increases the likelihood of arrhythmia-free survival six-fold compared to weight loss <10%.^11^ Similarly, pre-ablation weight loss improves long-term procedural outcomes.^18,19^ Furthermore, participation in a 6-month exercise-based intervention improves 12-month AF-free survival two-fold (40 vs. 20% in controls).^20^ In patients with sleep apnoea, CPAP therapy has been shown to reverse atrial remodelling,^21^ whilst the pro-arrhythmic effects of alcohol have also been observed in mechanistic studies, with acute exposure leading to shortened atrial effective refractory periods.^22^

Our survey revealed significant variation in respondents’ confidence levels in managing AF-related lifestyle and risk factors. Perhaps unsurprisingly, traditional cardiac risk factors, such as hypertension and smoking, were associated with the highest confidence levels, whereas risk factors more often managed by non-cardiologists—such as obesity, sleep-disordered breathing, and psychological distress—were identified as areas where respondents felt least confident. This gap in confidence is relevant, given the well-documented impact of these risk factors on AF progression and outcomes.^23–28^ Generally, lack of confidence, training, and resources appear to be a significant obstacle to managing these risk factors effectively.

Specifically, our data indicate that only a quarter (23.5%) of patients with AF and obesity are referred for formal dietary advice and that assessment and management of sleep-disordered breathing and psychological distress in patients with AF is suboptimal. Low referral rates for specialised services—such as for sleep studies or weight management/dietary advice—suggest that healthcare professionals may lack the resources or confidence to manage these complex conditions effectively. Similarly, lack of systematic screening for sleep-disordered breathing and psychological distress—which are not only common in AF patients but also associated with worse outcomes—is concerning, given the significant roles these conditions play in the pathophysiology of AF.^24,25^ Integrating validated screening tools for sleep-disordered breathing (e.g. the Epworth sleepiness scale^29^ and the STOP-Bang questionnaire^30^) and psychological distress (e.g. the GAD-7 questionnaire to screen for anxiety^31^ and the PHQ-9 questionnaire to screen for depression^32^) into routine AF care may enhance the management of these risk factors and ultimately lead to improved patient outcomes.

### Underutilisation of exercise-based cardiac rehabilitation

In patients with AF, exercise-based cardiac rehabilitation leads to improvements in exercise capacity, cardiac function, symptom burden, and health-related quality of life.^33–35^ Despite this, in our survey, respondents reported referring only 10% of patients with AF to such programmes. The inability of local programmes to accept patients with AF only (i.e. without another indication for cardiac rehabilitation), lack of patient motivation and engagement, and poor understanding of the local referral process were the most commonly identified barriers. Furthermore, in centres with access to cardiac rehabilitation, fewer than one in five programmes (17.9%) accepted direct self-referrals from patients. This highlights the need to expand the availability of exercise programmes tailored to AF patients and address structural limitations that prevent referral. Additionally, initiatives promoting patient engagement, through educational campaigns, reward schemes, and digital health tools may help overcome motivational barriers that limit uptake and completion of exercise programmes.^36,37^

### Systemic challenges to effective LRFM

Delivering comprehensive and meaningful LRFM in patients with AF can be challenging as it requires regular and intensive follow-up. A striking finding of our survey was that nearly 40% of respondents reported that their healthcare system is poorly designed to deliver meaningful LRFM in patients with AF. This suggests that, while LRFM is a cornerstone of AF management, its implementation is often hindered by systemic issues such as inadequate resources, poor inter-disciplinary coordination, and fragmented care models.

Furthermore, disparities between the current state of healthcare delivery and the perceived ideal model, as reported by respondents, underscore the need for structural reforms (*Figure 2B*). Specifically, the desire for a greater involvement of community-based clinicians and nursing staff in LRFM suggests that care models need to evolve from hospital-centric to more integrated, community-oriented approaches. Enhancing the role of primary care teams and providing appropriate training could help bridge this gap and facilitate the delivery of more comprehensive AF care.

Scientific guidelines—such as the 2024 ESC guidelines on the management of AF^14^—can assist clinicians in optimising patient care, although these are often poorly implemented. For example, integrating stroke prevention and rhythm control strategies into clinical practice is often complex, relying on a well-informed and collaborative team of healthcare professionals. The STEEER-AF trial (‘Stroke prevention and rhythm control treatment: evaluation of an educational programme of the European Society of Cardiology in a cluster-randomized trial in patients with atrial fibrillation’), a collaborative project between the ESC, EHRA, and ESC Council on Stroke, aims to assess the impact of a structured educational programme for healthcare professional on guideline adherence and clinical outcomes.^38^ The results of STEEER-AF will help enhance patient pathways, particularly through the development of a ‘patient version’ of the 2024 AF guidelines.^39^ Beyond this, targeted strategies seeking to improve patient engagement in underserved populations are critical, namely, rural, socioeconomically deprived and elderly communities.^40^

### Limitations

While our survey provides a novel perspective into current LRFM practices in AF management, we acknowledge its limitations. Importantly, the survey's reliance on self-reported data from a convenience sample of healthcare professionals introduces the potential for selection bias. Notably, the number of respondents varied significantly across countries, with a high percentage (21.3%) from a single country. Due to distribution via the EHRA network, the vast majority of respondents are cardiology healthcare professionals, specifically clinicians specialising in electrophysiology or cardiac devices. In clinical practice, many patients with AF never encounter such specialists and are managed by other healthcare professionals—namely, general practitioners—whose views are not accurately represented in this survey. Given the role of community doctors and nurses in the management of patients with AF, future studies may wish to explore collaborative approaches between hospital and community care settings to improve the care of patients with AF. Furthermore, the survey focused on respondents’ perceptions and self-reported practices, which may not align with actual clinical behaviour; observational studies or audits of clinical practice are needed to validate these self-reported findings and explore the true extent of LRFM integration in AF care.

## Conclusion

Our study provides valuable insights into current LRFM practices in patients with AF across EHRA countries, highlighting the variation and barriers in delivering effective LRFM. These barriers, which appear multifactorial, include both systemic challenges and gaps in healthcare professionals’ confidence. Addressing these will require coordinated efforts at both the healthcare system and provider levels, with a focus on improving access to specialised services, enhancing education and training for clinicians, and promoting a more integrated approach to AF care. Ultimately, overcoming these challenges is crucial in improving clinical outcomes for patients with AF and reducing the overall burden of the condition on healthcare systems.

## Supplementary Material

euaf075_Supplementary_Data

## Data Availability

The data underlying this article will be shared on reasonable request to the corresponding author.
