# Supplementary material for: Lifestyle and risk factor modification in atrial fibrillation: a European Heart Rhythm Association survey
Source: Europace. 2025 Mar 28;27(4):euaf075. doi: 10.1093/europace/euaf075 (PMC11983686; doi:10.1093/europace/euaf075)
Supplement: euaf075_Supplementary_Data [file euaf075_supplementary_data.doc]

**Supplementary Material – Appendix 1: Full survey**

| **Lifestyle and risk factor modification in atrial fibrillation – an EHRA survey** |
| --- |

**Introduction**

Dear colleague,

The European Heart Rhythm Association (EHRA) Scientific Initiatives Committee (SIC) is exploring the ways in which atrial fibrillation (AF) is managed by healthcare professionals from a lifestyle and risk factor modification perspective.

This survey includes questions on your experience of managing common lifestyle and risk factors in patients with AF, such as cardiac rehabilitation, obesity, sleep-disordered breathing, alcohol use, and psychological health.

On behalf of the EHRA SIC, we thank you for your participation, time and effort.

GDPR disclaimer:

Your participation is anonymous.

We will not disclose your identity to any third party.

We comply with the European General Data Protection Regulation (GDPR) 2016/679. Any personal data processed in connection with this survey will be treated confidentially and only used by the ESC for the purposes of market research and not for promotion. Survey results will be kept for a maximum of 48 months for analysis and quality control purposes. We take all reasonable care to prevent any unauthorised access to your personal data. We respect your privacy and your right to access, modify, or remove your personal data. At any time, you can ask to know what personal data is being held. If you have any questions about data protection or require further information, please contact our data protection officer (DPO) at [dpo@escardio.org](mailto:dpo@escardio.org).

You have the right to end your participation in this survey at any time.

**1. Please confirm that you have read the above and agree to participate in this survey.**

1. Yes
2. No

**Personal and professional information**

**2. In which country do you work?**

1. Drop-down list of EHRA countries

**3. Which of the following professional titles best describes you?**

1. Nurse working within a cardiology department
2. Nurse working outside of a cardiology department
3. Cardiology trainee/fellow
4. Cardiology consultant/attending having completed training in the last 10 years
5. Cardiology consultant/attending having completed training greater than 10 years ago
6. General practitioner/family doctor
7. Other (please specify)

(If answer to previous question is a, c, d or e): **4. What is your primary sub-speciality within cardiology? If you have more than one primary sub-speciality, select all that apply.**

1. Electrophysiology
2. Devices
3. Interventional cardiology
4. Heart failure
5. Cardiac imaging
6. Inherited cardiac disease
7. Adult congenital heart disease
8. I do not have a primary sub-speciality; I practice general cardiology
9. Other (please specify)

**Lifestyle and risk factor modification: general**

**In the following questions, ‘lifestyle and risk factor modification in AF’ refers to the management of hypertension, diabetes, physical inactivity, raised body mass index, sleep-disordered breathing, smoking, alcohol excess and psychological distress.**

**5. How well do you feel that the healthcare system that you currently work in is designed to deliver meaningful lifestyle and risk factor modification in patients with AF?**

1. Very well
2. Well
3. Neither well nor badly
4. Badly
5. Very badly
6. I am not sure

**6. In your current healthcare system, which group of healthcare professionals plays the leading role in encouraging and implementing lifestyle and risk factor modification in patients with AF? If the role is shared between more than one group, select all that apply.**

1. Hospital-based clinicians
2. Hospital-based nursing staff
3. Community-based clinicians
4. Community-based nursing staff
5. No clear leading role

**7. In an ideal healthcare system, which group of healthcare professionals do you feel would be best placed to encourage and implement lifestyle and risk factor modification in patients with AF? If you feel the role should be shared between more than one group, select all that apply.**

1. Hospital-based clinicians
2. Hospital-based nursing staff
3. Community-based clinicians
4. Community-based nursing staff
5. Other (please specify)

**8. How comfortable do you feel managing the following AF lifestyle / risk factors?**

Slider scale from 1 (Not at all comfortable) to 5 (Very comfortable) for each of the following:

1. Hypertension
2. Diabetes
3. Physical inactivity
4. Raised body mass index
5. Sleep-disordered breathing
6. Smoking
7. Alcohol excess
8. Psychological distress

**Exercise-based cardiac rehabilitation**

**9. In an average week, how many patients with AF do you manage? The term ‘manage’ refers to patients that you see or treat in any clinical setting (e.g. in the outpatient clinic, on the inpatient wards, and/or in the cardiac catheter laboratory).**

Free-text box accepting numerical answer only.

**10. Of the total cohort of patients with AF that you manage, what proportion do you believe would benefit from exercise-based cardiac rehabilitation?**

Slider scale from 0% to 100%

**11. In your current place of work, is it possible to refer patients with AF to an exercise-based cardiac rehabilitation programme?**

1. Yes
2. No
3. I am not sure

**12. Of the total cohort of patients with AF that you manage, what proportion do you believe are actually referred for exercise-based cardiac rehabilitation?**

Slider scale from 0% to 100%

(If yes to 11): **13. If a local exercise-based cardiac rehabilitation programme exists for patients with AF, which individual(s) is(/are) able to refer to this directly? Select all that apply.**

1. Hospital cardiologist
2. Hospital physician (non-cardiologist)
3. Community general practitioner/family doctor
4. Nurse specialist
5. Non-specialist nurse
6. Patient (self-referral)
7. I am not sure
8. Other (please specify)

**14. In your opinion, what is the most important barrier to patients with AF taking part in an exercise-based cardiac rehabilitation programme? Select only one.**

1. Poor understanding of the local referral process
2. Local cardiac rehabilitation programme does not accept patients with AF only
3. Associated financial cost
4. Lack of patient motivation / engagement
5. Presence of symptoms preventing exercise
6. Excess weight hindering exercise
7. In my opinion, there are no systemic barriers
8. Other (please specify)

**Raised body mass index (BMI)**

**15. Do you (or your department) use a strict BMI cut-off when deciding on suitability for AF ablation?**

1. Yes
2. No

(If yes to above): **16. What BMI cut-off do you use when deciding on suitability for AF ablation?**

Answer: Drag bar from BMI of 20 to 70 kg/m^2^

**17. What percentage of patients with AF and raised BMI do you refer for formal/comprehensive dietary advice (e.g. by a qualified nutritionist or dietitian)?**

Slider scale from 0% to 100%.

**18. In your opinion, should arrhythmia healthcare professionals (e.g. arrhythmia doctors and/or nurses) regularly caring for patients with AF be competent in prescribing weight loss medications (e.g. orlistat, liraglutide, semaglutide)?**

1. Yes
2. No – this should be reserved for healthcare professionals with detailed experience of weight management
3. I am not sure

**19. Do you have any experience in prescribing weight loss drugs (e.g. orlistat, liraglutide, semaglutide) to patients with AF?**

1. Yes – lots of experience
2. Yes – some experience
3. No

**20. In your opinion, which one of the following options is the biggest barrier to weight loss in patients with AF?**

1. A systematic lack of health care structure and support
2. Presence of symptoms preventing exercise
3. Patient lack of nutrition knowledge
4. Lack of patient motivation / engagement
5. There are no barriers
6. Other (please specify)

**Sleep-disordered breathing**

**21. Which of the following statements best describes your practice of assessing for sleep-disordered breathing in patients with AF?**

1. I systemically assess all/almost all patients with AF for sleep-disordered breathing, irrespective of the clinical picture
2. I only assess patients with AF for sleep-disordered breathing if the clinical picture is suggestive of possible sleep-disordered breathing (e.g. if the patient has a raised BMI)
3. I sporadically assess patients with AF for sleep-disordered breathing, whether or not the clinical picture is suggestive of sleep-disordered breathing
4. I do not assess patients with AF for sleep-disordered breathing

**22. In patients with AF whom you deem to be at risk of sleep-disordered breathing, do you complete a validated sleep-disordered breathing questionnaire?**

1. Yes – all of the time
2. Yes – most of the time
3. Yes – some of the time
4. No – never

**23. If symptom enquiry, clinical examination, or validated questionnaires are suggestive of sleep-disordered breathing, what should an arrhythmia healthcare professional’s (e.g. arrhythmia doctor or nurse’s) next step most commonly be?**

1. Direct referral for sleep studies
2. Direct referral to sleep / respiratory specialist
3. Ask general practitioner / family doctor to consider further investigation or referral as appropriate
4. I am not sure
5. Other (please specify)

**24. In patients with AF and known sleep-disordered breathing on continuous positive airway pressure (CPAP) therapy, how often do you stress the importance of compliance with CPAP?**

1. Never
2. Sometimes
3. Most of the time
4. Almost every time
5. Every time

**Alcohol excess**

For the following section, 1 unit of alcohol is equivalent to 8 grams of pure alcohol, and is generally equivalent to 1 shot of spirit, half a glass of beer, or a small glass of wine.

**25. Do you usually ask your patients with AF about their alcohol intake?**

1. Yes
2. No

**26. What percentage of patients that you manage with AF do you believe drink to excess?**

Slider scale from 0% to 100%.

**27. What is the maximum number of units of alcohol that you would usually advise a patient with AF to drink per week?**

1. 0 units
2. Less than 5 units
3. 6 to 10 units
4. 10 to 14 units
5. 15 to 20 units
6. A value greater than 20 units
7. I do not advise on a strict unit cut-off
8. Other (please specify)

**28. In your current place of work, are you able to refer patients with AF and alcohol excess to a dedicated alcohol reduction/cessation service?**

1. Yes
2. No

**Psychological health**

**29. In your opinion, what percentage of patients with AF experience psychological distress (e.g. anxiety or depression)?**

Slider scale from 0% to 100%.

**30. How often do you assess for psychological distress (e.g. anxiety or depression) in patients with AF?**

1. Never
2. Sometimes
3. Most of the time
4. Almost every time
5. Every time

**31. Which of the following statements best describes your assessment of psychological distress in patients with AF?**

1. I never assess my patients with AF for psychological distress
2. When I assess patients with AF for psychological distress, I most often rely on informal questioning regarding their symptoms and lived experience
3. When I assess patients with AF for psychological distress, I most often rely on the use of validated psychological health questionnaires
4. When I assess patients with AF for psychological distress, I most often rely on a combination of informal questioning and validated psychological questionnaires
5. Other (please specify)
